# Promoting the Resilience of the Italian Population Against SARS-CoV-2

**DOI:** 10.3389/fpsyt.2020.560017

**Published:** 2021-02-16

**Authors:** Anna Giulia Bottaccioli, David Lazzari, Francesco Bottaccioli

**Affiliations:** ^1^University “Vita e Salute” San Raffaele, Milan, Italy; ^2^Italian Society of Psychoneuroendocrineimmunology (SIPNEI), Rome, Italy; ^3^National Council of the Order of Psychologists, Rome, Italy; ^4^Department of Life, Health and Environmental Sciences, University of L'Aquila, L'Aquila, Italy

**Keywords:** immune system, pandemic, COVID-19, mental health, stress, resilience

## Abstract

The COVID-19 pandemic, due to its exceptional level of impact on the populations of the richest and most technologically advanced nations—which are experiencing unprecedented widespread mortality, fear, and social isolation—and due to the considerable difficulties faced by health services in coping with the emergency and the uncertainty regarding the evolution of the pandemic and its foreseeable heavy economic repercussions on a global scale, requires a change in the approach to the prevention and treatment of the infection based on the integration of biomedical and psychological sciences and professions. A survey of the Italian pandemic population, the results of which we report here, shows a widespread state of psychological distress, which, based on decades of scientific and clinical evidence on the relationship between mental states and immune system efficiency that we summarize in this paper, plausibly weakens the resistance of individuals and the population to SARS-CoV-2 infection. Italy can deploy a great force, represented by tens of thousands of psychologists and psychotherapists, who, as health workers, could be employed, alongside local and hospital medicine, in primary care and in promoting the resilience of citizens and health workers themselves, who are subject to a deadly work stress that also includes a widespread threat to their lives.

## Epidemiology of SARS-COV2 Infection

Almost 500 years ago, in 1546, Italian doctor Girolamo Fracastoro (1) proposed, for the first time, the interhuman transmission of pathogens (which he called *seminaria*) as the cause of epidemic infections (contagion). The COVID-19 pandemic is caused by the interhuman infection of a pathogen, a new coronavirus, classified as SARS-CoV-2 by the International Committee on Taxonomy of Virus (2) due to its similarity to SARS-CoV, which gave rise in 2002 to the severe acute respiratory syndrome (SARS) epidemic in the Chinese province of Guangdong. In December 2019, SARS-CoV-2, in Wuhan, China, caused an epidemic (3) that, in January 2020, affected the whole of the province of Hubei and that, as of February, affected Lombardy, Veneto, Emilia Romagna, and subsequently the whole of Italy.

Currently, confirmed cases of coronavirus disease (COVID-19) are on the rise in Europe and the United States and virtually the entire planet (4). Its fatality rate, which varies from country to country and presents high levels of uncertainty and likely underestimation, is, in any case, higher than the initial forecasts: It ranges from just over 5% of confirmed infections declared by China to more than 17% recorded in Lombardy, which alone accounts for ~50% of all Italian mortality from COVID-19. However, researchers and local administrators put forward the hypothesis that the numbers of infected people and deaths are much higher than official estimates in both China and Italy, particularly in the most affected areas, such as in the provinces of Bergamo and Brescia in Lombardy (5). The elderly, who most frequently have comorbidities, and the male gender are the most affected groups. In Italy, the mortality rate from COVID-19 as of September 2020 recorded by the Istituto Superiore di Sanità, concerned 57.4% men and 42.6% women. The fact that, in the 60–69 age group, the male mortality rate is even lower for women is significant. Last, according to this survey, 49% of the deceased were 80 years of age or older (6). According to another survey by the Istituto Superiore di Sanità carried out on Residenze Sanitarie Assistenziali and other nursing homes for the elderly the facilities in Lombardy are particularly affected by the epidemic: It is estimated that, in March, more than half of the mortality recorded in these centers is attributed to COVID-19 (7). The United States, Spain, Italy, France, England, and Germany are the most affected countries, but the infection is rapidly spreading globally (https://coronavirus.jhu.edu/map.html). SARS-CoV-2 is more invasive than other human coronaviruses (SARS-CoV and MERS-CoV, responsible for SARS and Middle East respiratory syndrome outbreaks, respectively), likely due to mutations in gene sequences coding for the viral spike protein (8), which made it more suitable for interaction with the cellular protein angiotensin-converting enzyme 2, which acts as a gateway for the virus in cells of the human respiratory mucosa.

### The Mental States of the Population During Lockdown

The COVID-19 pandemic, which, unlike SARS and MERS, was not limited to some eastern areas (China and the Middle East), affecting the heart of the West, has generated astonishment and disbelief among the population. In the imagination of the Western citizen, epidemics were a memory, mostly literary, of the past and, in the present, a phenomenon of the most unfortunate areas of the world, living without hygiene, food, or medicine. In this case, however, the epidemic is a dramatic reality of the wealthy, technological West, the cradle of scientific medicine. In addition, the infection usually has a trivial onset with fever, cough, sore throat, and asthenia, symptoms that everyone has experienced many times during their lives without serious consequences. However, in the pandemic context, the subject who experiences them can interpret them in a much more threatening way, like the beginning of a chain that, quite quickly, can lead to a serious disease condition. For these and other reasons, the new epidemic reality is still struggling to be mentally processed by people, upon whom, above all, weigh the restrictive measures that, moreover, the governments of Western countries themselves have taken with great reluctance and wavering attitudes (in particular the American and English governments).

### The Psychosocial Distress of the Italians During Lockdown

Before the pandemic, according to international studies that also include Italy, the prevalence data of the most common mental disorders were in a range of between 4 and 6.7% for anxiety and between 4 and 5.4% for depression (9, 10). The Italian Central Institute of Statistics (11) notes the prevalence of these problems in a “Multi-purpose survey of Italian households” limited, however, to subjects aged between 16 and 65 years. In this context, it is found that 5.14% of respondents reported current depressive symptoms, and 3.6% reported symptoms of anxiety. After the announcement of the nationwide lockdown in Italy on March 9, 2020, the stress index score trended toward the higher scores as shown in the survey carried out by the Piepoli Institute for the National Council of the Order of Psychologists (April 8, 2020) (see Table 1). Data on the main sources of distress reveal high levels of anxiety, sleep disorders, irritability, depressed mood, relationship problems, and eating problems (see Table 2).

**Table 1:** Stress index score variation among adult population before and after the announcement of the nationwide lockdown in Italy.

**Stress index score**	**Date of detection: 3/2/20**	**Date of detection: 3/9/20**	**Date of detection: 4/6/20**
High	27%	43%	41%
Medium	45%	45%	38%
Low	28%	12%	21%

*The survey was carried out by the Piepoli Institute for the National Council of the Order of Psychologists (April 8, 2020) on a total sample of 501 subjects representative of the Italian population. On 9 March 9, 2020, the government of Italy extended lockdown to the entire country: This raised the stress index national trend toward the higher scores, and it remained high after 1 month. The stress index score is a 10-point scale on which 1 is little or no stress and 10 is a great deal of stress. The indicator was calculated by transforming the stress scale from 1 to 10 points into a percentage from 1 to 100%, and the score obtained was then classified as low (1–40%), medium (41–70%), or high (71–100%)*.

**Table 2:** Main forms of psychological distress.

**Psychological distress**	**Total**	**Men**	**Women**
Stress/Anxiety	42%	40%	43%
Sleep disorders	24%	19%	28%
Irritability	22%	21%	22%
Depressed mood	18%	15%	21%
Eating problems	10%	10%	10%
Problem in relationships	7%	6%	8%
Problem with partner	3%	5%	5%
Problem with sons	1%	5%	1%
None	28%	31%	25%

*The survey was carried out by the Piepoli Institute for the National Council of the Order of Psychologists (April 8, 2020) on a total sample of 501 individuals representative of the Italian population divided by gender*.

The majority of Italians (80% of interviews) attribute these forms of distress to the pandemic (34% greatly and 46% fairly), a percentage that has increased from the beginning of the survey to date: on February 24, it was 62%. Since the beginning of the COVID pandemic, 67% of Italians say that their level of distress has increased. The sources of this distress are linked to the specific restrictions and living conditions (51% social, 27% not being able to do outdoor activities, 24% having little space available, 20% not being able to go to work, 9% forced cohabitation), 58% declare distress associated with financial prospects, and 31% are worried about their worsening psychological experience. What emerges is a sharp increase in psychological distress, which is based not only on the conditions/restrictions of the pandemic (51%) but also—and increasingly—on concerns regarding social and financial prospects (58%).

A recent European survey conducted in three countries (Italy, Spain, and the United Kingdom) confirms that there is a widespread state of psychological distress in the general population. Predictive analyses show that the mental health of a large proportion of the population is at high risk for stress, anxiety, and depression in these countries (59, 67, and 57%, respectively; see Table 3) due to socioeconomic vulnerabilities and worsened conditions since the pandemic onset (12). An Italian study conducted between the end of April and the beginning of May 2020, which followed other research carried out in the first phase of the lockdown, documents the persistence of high levels of stress and depression, particularly in people with preexisting stressful conditions, especially young people and people with poor ability to cope with difficulties and with tendencies toward isolation and lack of motivation (13). A Chinese study, conducted after a month of lockdown, reported an increase in cases of post-traumatic stress disorder with a prevalence in women (14).

**Table 3:** Percentage of depressed subjects during the pandemic among adult population in Italy, Spain, and the United Kingdom.

**Total**	**Italy**	**Spain**	**United Kingdom**
61%	59%	67%	57%

*The survey was carried out by Open Evidence, a spinoff of Universitat Oberta de Catalunya (UOC), realized with the contribution of BDI- Schlesinger Group and Università degli Studi di Milano, Universitat Oberta de Catalunya, Universidad Nacional de Colombia, Università di Trento, University of Glasgow, on a total sample of 1,000 subjects divided by the countries involved in the survey. The percentages refer to those who reported to have been depressed in the last 7 days (reference period: from 4/24/2020 to 5/1/2020). The Depression, Anxiety and Stress Scale (21 Items) and Stanford Acute Stress Reaction Questionnaire were used to quantify depressive symptoms*.

The additional surge in infections in the United States, Brazil, India, and Europe after summer 2020 could potentially worsen mental illness in large sectors of the population. Particular attention should be paid to COVID-19 survivors who present anxiety, depression, sleep disorders, loss of memory, and quality of life deterioration in significant percentages after months post-discharge from hospital (15). The health emergency involves the psychological well-being of both individuals (e.g., insecurity, confusion, emotional isolation, stigma) and communities (concerns regarding the economy, work, school, treatment, etc.), triggering widespread situations of psychological distress (which sometimes lead to psychiatric disorders), negative behavior, and non-compliance with safety guidelines (16). Alongside this widespread distress are the more specific forms of distress and disorders present in people with COVID-19 who are hospitalized or in quarantine at home, the relatives of sick and deceased people, and health workers and those no longer exposed to the pandemic who have been the subject of initial international studies (17).

## What do we Know About the Pathogenesis of COVID-19?

The majority of individuals who have come into contact with SARS-CoV-2 have few or often no symptoms. In a smaller proportion of those infected—we do not know exactly in what proportion—the infection can evolve into an interstitial pneumonia that can give rise to acute respiratory distress syndrome (ARDS) with a possible fatal outcome. The virus is transmitted to humans through droplets and aerosols and other routes of transmission have been reported, such as contact with contaminated surfaces, especially plastics (18). A fecal–oral transmission route is also reported based on the identification of RNA or live infectious SARS-CoV-2 in feces of some Chinese patients with COVID-19 (19).

From the upper airways, the virus, if not contained, spreads to the bronchi and lungs and then to the intestines and other organs, especially the kidney, heart, and brain. The vascular system is fully affected with altered coagulation and the formation of clots, which can also give rise in a proportion of cases (20) to disseminated intravascular coagulation (21). The severity of the disease depends on the level of systemic inflammation and the degree of involvement of the lungs, which may present on computed tomography as “patchy ground-glass opacities” (17) and other signs affecting the pleura (22). In the early phase of the disease, non-specific clinical symptoms occur: fever, cough, and dyspnea as well as immune changes, such as a high neutrophil–lymphocyte ratio (23), functional exhaustion of T cells (24), and overproduction of IL-1β and IL-6 and in contrast low production of IFN-γ (25). If the disease evolves into a more severe form alongside these alterations, there are other signs of systemic inflammation, particularly in the vessels: a condition produced by a so-called “cytokine storm,” i.e., a high concentration of inflammatory cytokines released by immune cells and also by other damaged cells. In patients who develop more severe forms of the disease, SARS-CoV-2 is able to evade the immune response that could block it, which is based on a T helper type I (Th1) response and activated CD8+ cytotoxic T lymphocytes. Instead of this antiviral immune circuit, there is an increase in the activity of neutrophils. Neutrophilia and lymphopenia on complete blood count tests seem to be a consistent feature of COVID-19. Neutrophil activity, if not accompanied by the involvement of B lymphocytes together with cytotoxic T lymphocytes and the Th1 circuit, not only does not eliminate infection, but may also be at the origin of the state of hyperinflammation frequently observed in the advanced disease, which characterizes the clinical transition from pneumonia to ARDS and the overproduction of inflammatory biomarkers, such as cytokines and neutrophil-derived extracellular traps (NET). The NET formation, a phenomenon discovered and studied in recent years, reflects the ability of neutrophils to expel their DNA and create extracellular networks composed of chromatin fibers, cytosolic and granule proteins, and inflammatory and oxidizing substances that are able to trap and destroy pathogens (26). NET formation, when unregulated, is a key factor in the production of a highly inflammatory state (27). The involvement of NET scaffolds in autoimmune vasculitis and systemic lupus erythematosus (28) as well as in ARDS in the context of SARS-CoV–related pneumonia has been documented (29). It is not difficult to assume that NET formation also is likely involved in advanced COVID-19; findings from postmortem series of direct autopsies conducted on patients who died from COVID-19 demonstrate thrombi and neutrophilic plugs in the lungs, heart, kidneys, liver, spleen, and brain (30).

## A PNEI Approach to Identifying the Main Factors of Resistance to Infection and Mass Resilience During the Pandemic

The vast majority of the population has endogenous resources to fight the infection, which can be silent or with few symptoms. The psychoneuroendocrinoimmunological (PNEI) approach, which studies the two-way relationships between the psychic dimension and biological systems in the environmental and social context (31, 32), provides an appropriate model for the identification of risk factors and resistance to infection. It also describes how to understand the effects of infection on an affected person's overall health, including the person's mental state (33).

As described, the central issue is the balanced immune response to SARS-CoV-2. A number of factors can regulate or unbalance the antiviral response, including both individual and group factors. Individual factors related to diet, physical activity, stress, and mental states are discussed in the next paragraph. Here, we briefly point out the effects of air pollution on lung inflammation and the development of COVID-19.

Recent research by the European Environment Agency estimates that PM2.5 pollution in 2016 was responsible for ~412,000 premature deaths in Europe caused by heart attacks, strokes, and lung disease (34).

It is demonstrated that chronic exposure to air polluted by fine particulate matter (i.e., PM10, PM2.5, and ultrafine PM <0.1 matter) causes damage to the respiratory system. These by-products of combustion derived principally of fossil fuels, mainly PM2.5 matter, can penetrate the bronchial and pulmonary tract, and ultrafine PM <0.1 particulate matter can pass directly into the bloodstream and spread to the organs. The alteration of the respiratory defensive systems caused by PM2.5 concerns damage to the mucosal barrier, respiratory microbiota, and immune cells (35). This immune dysregulation can be a determining factor for serious respiratory diseases, such as lung cancer and chronic obstructive pulmonary disease, or can cause chronic low-grade inflammation of the upper and lower airways by activating granulocytes (neutrophils) and mast cells in the respiratory mucosa and macrophages in the alveoli and lung interstices. In turn, chronic low-grade inflammation can promote the pathogenic action of various respiratory bacteria and viruses, including SARS-CoV-2.

A research study in progress at Harvard University, in the Department of Biostatistics, School of Public Health, recorded a direct relationship between PM2.5 particulate matter exposure to air pollution and COVID-19 mortality in the United States. Harvard epidemiologists find that the increase of just 1 μg/m^3^ in PM2.5 is associated with a 15% increase in mortality rate from COVID-19 (36). An intriguing and disturbing fact is the overlap between levels of fine particulate pollution in Lombardy and the incidence of COVID-19, which is highest in areas of maximum air pollution, which has been particularly high in the last two decades. At the beginning of January 2020, at the time when the contagion was thought to be beginning to spread, the Lombardy Environmental Protection Agency reported very high concentrations of “PM10 of up to 180 μg/m^3^, i.e., 3.6 times the legal limit for several consecutive days in different areas of Lombardy, including Milan” (37).

Obviously, fine particulate pollution is not the only factor explaining the exceptional mortality rate recorded in some provinces of Lombardy; there are other factors, on which in-depth public health investigations are expected to be conducted. The fact remains that, if the spread of the infection is to be effectively countered by increasing the population's defensive capacity toward SARS-CoV-2, particularly during the phase of resuming work, the abatement of air pollution is a major antiviral measure as well as for containing the circulation of the infection.

## Supporting the Equilibrium of the Immune System

The immune system is influenced by several factors, including diet, physical activity, psychological state, and air and environmental pollution. We have just dealt with the latter aspect; therefore, let us briefly look into the others.

### Nutrition and Microbiota

The severe form of COVID-19 with ARDS and cytokine storm syndrome is more frequently observed in patients with diabetes, hypertension, cardiovascular disease, and obesity due to a preexisting systemic chronic inflammation and pro-inflammatory cytokine hyperproduction (mainly IL-6).

Hyperglycemia, often observed in cases of stress and infection, has been reported in 51% of patients with COVID-19 (25). It is also demonstrated that a condition of hyperglycemia and high lactate production reduces innate immune type I interferon (IFN I) production, weakening the host defense against viruses (38).

A diet low in protein is one of the main causes of immunodeficiency in the elderly population (39), and the lack of an adequate pool of amino acids has been associated with low production of immunoglobulins, thymic atrophy, reduced proliferation of naive lymphocytes, and poor cell maturation with lytic activity (natural killer and lymphocytes with cytotoxic activity). Adequate intake of essential micronutrients at all stages of life contributes significantly to the proper maturation of the immune system and efficient response to infection. There are numerous studies on the specific effects of essential micronutrients on the functioning of the immune system: Monounsaturated fatty acids (oleic acid), B group vitamins, fat-soluble vitamins (A, D, E), beta-carotene, iron, copper, zinc, and selenium have gathered the most evidence (40).

As is well-known, diet selects and profoundly shapes the microbiota, a complex set of resident microbial populations (bacteria, viruses, and fungi) that form colonies in contact with the mucous membranes of the body and, therefore, also in the respiratory mucosa. A state of dysbiosis, which can occur as a result of several conditions, including the use of drugs (antibiotics, antacids), an inflammatory diet, surgery, and hospitalization, can be associated with several life-threatening infectious conditions: infection with multiresistant germs, pseudomembranous colitis from *Clostridium difficile*, or sepsis (39).

On April 4, 2020, the Italian Scientific Societies of Clinical Nutrition and Anesthesia-Resuscitation published a joint document (41) in which they drew up recommendations for the nutritional treatment of patients affected by COVID-19 and hospitalized in intensive and subintensive care units in Italian hospitals. The clinical features of COVID-19–critical patients show widespread malnutrition. Malnourished COVID-19 patients in intensive and subintensive care are associated with higher hospital costs, prolonged stays, and increased mortality. The Faculty of Medicine of Zhejiang, in its “COVID-19 Disease Therapy and Prevention Manual” released in March 2020 (42), includes nutritional therapy and the use of probiotics in the standard of care of hospitalized patients in order to reduce the rate of bacterial superinfections, reduce hospitalization in intensive care, and accelerate functional organ recovery. The early initiation of nutritional therapy is, therefore, vital, particularly in patients with organ failure and sepsis, but could significantly change the course of the disease even in non-critical patients hospitalized on ordinary wards or treated at home (43).

### Physical Activity

One of the main effects of forced quarantine during a pandemic is reduced mobility. Although everyone may suffer from a prolonged period of almost total physical inactivity, the elderly population may, once again, pay the highest price. Reduced mobility in the elderly (44), in fact, raises the fragility index dangerously upward, rapidly depletes muscle reserve, and accelerates bone turnover, promoting sarcopenia, osteo-articular degeneration, repeated falls, and osteoporotic fractures; it worsens respiratory function, increasing the risk of acute seasonal airway diseases and chronic bronchopathies, and alters metabolism and blood pressure regulation, increasing the use of specific drugs and, therefore, health expenditure. Regular physical activity is also a trophic stimulus for the brain; i.e., it effectively counteracts neurodegeneration (in key brain areas, such as the hippocampus) and the onset of dementia. An elderly person with reduced mobility who is hospitalized leads to an exponential increase in the burden of care and an increase in the average length of hospitalization and related pathologic consequences (bedding syndrome, bedsores, infectious risk, sarcopenia, worsening of cognitive functions, or risk of delirium or psychotic dissociation).

Regular physical activity also modulates immune function, making the response against viruses and cancer cells (Th1 circuit) more efficient. A significant example is studies on women with breast cancer (45) in which a change in immune profile (increase in natural killers and CD8+ lymphocytes) was recorded in response to a regular physical activity program. It has been demonstrated in several studies on elderly subjects (46) that it is mainly moderate aerobic exercise that counteracts immunosenescence, reducing inflammatory cytokines, such as TNF-α and IL-6; increasing the anti-inflammatory IL-10; and increasing the number of CD4+ and CD8+ lymphocytes and regulatory T lymphocytes. Physical activity also slows down cellular aging, which is at the root of many chronic diseases among the elderly, measured by the length of the telomeres (the end of the chromosomes) of the immune and skeletal cell genes of well-exercised elderly subjects (47).

### Stress and Its Regulation

During a pandemic, quarantine is in itself a highly stressful situation, in which several factors fuel psychological suffering: prolonged social isolation, fear of infection, sense of frustration, boredom, inadequate support, inadequate information, financial loss, and social stigma. Recent work published on the Chinese population (48) shows that there is a high level of psychological stress, anxiety, and depression and a lower quality of life in people affected by COVID-19 and in close family members than in those not directly affected. Family and social support and access to accurate and comprehensive information on one's own health and that of the community as well as clear communication on precautionary measures to be taken greatly reduce the stress load and risk of developing anxiety-depressive illness (49). A condition of prolonged stress brings profound adaptive changes in the psycho-neuro-endocrine-immunity network (32, 50): The psychological state is dominated by anxiety, depression, altered sleep–wake rhythm and anhedonia; the biological side is characterized by altered hypothalamic-pituitary-adrenal axis response and release of awakening and stress-induced cortisol, unbalanced activation of autonomic nervous system and increased adrenergic tone, pathological alterations in metabolic and cardiovascular functions, immune dysregulation, and a systemic and central inflammatory state.

Several experimental research studies focus on the two-way relationship between mental state, immune system, and inflammation, the latter defined as a silent killer at the basis of many current chronic degenerative diseases with high mortality (cardiovascular diseases, cancer, diabetes mellitus). Social isolation, low socioeconomic status, loneliness, previous trauma, or current living conditions dominated by fear or violence not only shorten life expectancy and increase morbidity for the most common chronic diseases, but are also associated with a higher level of inflammation (51) and lead to or aggravate the depressive state. In addition, the progressive increase in average age in itself constitutes a risk for the onset of depression, cognitive decline, and reduced self-sufficiency.

Aging is a physiological process that is accompanied by progressive alterations in immune function, dominated by reduced specific immunity response and non-specific increase in inflammation *(inflammaging*) (52). Loneliness (53) and social isolation (54), conditions that certainly affect many older people and are increasingly affecting larger sections of the population, emerge as independent and synergistic predictors of morbidity and mortality as well as the best known disease risk factors (55).

The depressive state correlates positively with an increased concentration of inflammatory molecules (cytokines). Abnormal inflammation appears clearly in a subset but not in the whole population of those suffering with depression. This subset is more resistant to psychological treatment (56). Depression, in these cases, can, therefore, be seen as a form of low-grade inflammation (57) particularly active in brain circuits involved in adaptive behavior and emotional state processing (limbic and prefrontal cortex); this leads to a pathological condition that, in its chronicization, is continuously fed by inadequate lifestyle behaviors (i.e., high sugar and fat diet, alcohol and illicit drug consumption, smoking, sedentariness, social avoidance) that, in turn, support the inflammatory state and worsen the psychological state in a vicious circle (33).

A recent population study (58) conducted on 24,325 Italian citizens living in Molise investigated the correlation between aspects of psychological health, such as the degree of resilience; depressive symptoms, and quality of mental life and an aggregate index of low-grade inflammation (the INFLA score) that measures the blood concentration of C-reactive protein, platelet count, and white blood cell concentration (neutrophil–lymphocyte ratio). The results of the study, conducted on a healthy population, showed higher INFLA scores in subjects with higher scores for depression, and the opposite condition, that is, reduced inflammation, was observed in the case of a high score for mental well-being. The correlation between INFLA score and depression is even more significant if lifestyle is associated, i.e., with a positive history for cigarette smoking, poor adherence to the Mediterranean diet, overweight/obesity, and reduced physical activity. The INFLA score also demonstrates, in depressed patients, a vigorous activation of innate immunity (high number of neutrophils, activation of monocytes, high neutrophil–lymphocyte ratio), increased synthesis of inflammatory cytokines and reduced lymphocyte activation.

The results are in line with a recent study of genetic analysis (59) that demonstrates the activation of at least 165 genes in major depressive disorder in humans, 90 of which are hyperexpressed mainly in cells of the innate immunity (neutrophils, monocytes, dendritic cells) and regulate through stable epigenetic changes inflammation and immune response. The pathophysiological interplays in the psyche-brain-immune network are particularly at risk among the elderly population in this historical era characterized by sudden and destructive environmental factors, such as the pandemic and the resulting state of isolation due to quarantine.

Several COVID-19 patient-management guidelines have highlighted the need to protect the mental health of citizens affected by the pandemic. Starting from the indications given by the WHO (60), several strategies have been identified to counteract the growing psychological distress. Yoga, mindfulness meditation and relaxation, and breathing exercises are the most commonly mentioned (61) and recommended techniques given that they are safe, free from side effects, and applicable in any emergency context from COVID wards to the homes of isolated patients (62). There are numerous randomized controlled trials conducted in elderly individuals, neoplastic and immunodepressed patients, or subjects at high cardiovascular risk, which have documented a statistically significant reduction in serum inflammation markers (C-reactive protein, cytokines) (63) and an enhancement of natural antiviral and anticancer immunity (increase in number and activity of natural killer cells) and the Th1 immune circuit among meditating subjects compared with controls (64).

## Promoting Psychological Resilience as Part of an Integrated Medical and Psychological Approach as a Means of Combating the Spread of the Epidemic

The real challenge is to intercept the widespread psychosocial distress and the more structured psychiatric disorders and provide an appropriate and comprehensive response.

The widespread nature of the emergency requires a stratification of the population in relation to the type of problems and consequent level of assistance. However, on the basis of Italian experience and international literature, the following areas can be hypothesized in potentially descending order:

1. The “front line”: People ill with COVID-19 confined at home and hospitalized, family members, bereaved people, the first-line health workers.
2. The “second line”: people in quarantine and people not affected by COVID but who are particularly at high risk due to physical or psychological frailty, such as people with chronic pathologies, intellectual or physical disabilities, psychiatric problems, lonely elderly people, workers in critical areas.
3. The “inner front” formed by the tens of millions of healthy people at risk of home confinement.

Faced with such a large potential target, it is necessary to deploy an integrated strategy that provides various options that have as their first objective to intercept the needs and provide answers.

- For the first line, a specific action must be guaranteed especially in hospitalization contexts with three main targets: infected ill people, their relatives, and health care workers. About the last category the surveys show high levels of depression, anxiety, sleep disorders, and distress, especially among the female gender and those working on the front line (65). In this context, it is necessary that the psychologists recruited are present in health care contexts and that their action is coordinated with the overall health care action. This includes remote home care for COVID-sick and non-hospitalized patients.
- The second line has intermediate needs compared with the above lines and includes all situations of people in quarantine, those suffering from physical or mental disorders, carriers of situations of fragility, or those requiring special support.
- For the extensive inner front, it is necessary to start from widespread proactive strategies of prevention and promotion of resources (at the social, community, group, and individual levels) and from the provision of psychological skills within the facilities with the greatest cost–benefit impact of the social network: primary care, regional health care services, social services, schools, community contexts, world of work. This network must guarantee an initial and widespread response—with remote or onsite methodologies—in terms of primary and secondary prevention as well as selecting and facilitating second-level interventions (e.g., for more structured and severe psychiatric diseases of psychopharmacological type).

The subgroups to which attention must be paid include the prison population, people with disabilities and their families, children with disorders or family problems, women in the peripartum period, and lonely elderly people. There is also the problem of support for workers who fall into situations of greater or lesser exposure to risk in relation to the type of work and who generally suffer from problems related to organizational changes (e.g., flexible working) and employment prospects.

As highlighted, there is a need for a program of initiatives that, proceeding from the widespread front to the front line, involves a shift from large-scale forms of primary prevention (e.g., dissemination of psychoeducational advice), promotion of resources and empowerment, listening, information, and telephone guidance to more targeted forms of intervention, such as psychological support, stress management, or psychotherapy in the form of remote or face-to-face assistance.

Given that in Italy there are more than 100,000 members of the Order of Psychologists classified as health professionals, half of whom specialize in psychotherapy, there are qualified professional resources to implement this strategy, and they are able to implement the current small number of psychologists structured in the Italian National Health Service (~6,500).

The activation of an initial-level psychological network, structured within the main health care hubs (GPs, regional services) as well as in support of schools, social services, and work contexts, is fundamental to intercept and respond to the widespread need.

Psychological interventions should assess and monitor COVID-related stress (e.g., exposure to infection, sick or deceased relatives), secondary adversities (e.g., financial problems), psychosocial effects (anxiety, depression, psychosomatic problems, sleep disorders, situations of conflict, and violence), and vulnerability indices (e.g., social conditions, preexisting psychophysical conditions) (16).

In this context, the initial level can proceed to dispatch any need for primary (GP, nurse), social, or other care that may be intercepted and act as a filter for the activation of second-level health interventions, such as mental health or neurodevelopmental disorders.

As regards remote psychological services, guidelines have been issued by the National Council of the Order of Psychologists (66), and for integrated medical and psychological teleassistance services for people in quarantine or in situations of special needs, there are indications from the Istituto Superiore di Sanità based on a triple stratification of care needs (67)

A particular issue is that of work stress and the high risk of burnout among health care workers. Suffice it to think of the number of deaths among the health professionals in Italy (more than 200) and the request of the President of the Italian Nurses to the Minister of Health to solicit urgent psychological help for these professionals. In relation to this emergency, the Italian National Institute for Occupational Accident Insurance (INAIL) in collaboration with the Order of Psychologists has prepared a methodology of psychological intervention for health care workers (68).

There is currently an overall difficulty in launching a strategy capable of articulating and integrating psychological intervention into health care and the social network, both due to the forced priority given to medical assistance emergencies in the first phase and, above all, due to a widespread cultural problem that tends to separate psychological aspects from health-related issues and health interventions in general—a cultural problem that does not seem to concern the Italian population, yet which, on the contrary, is very favorable to a greater presence of psychologists in primary care services (i.e., hospitals, nursing homes, social services, family physicians' offices) in order to counteract the COVID-19 pandemic effects (Piepoli Institute for CNOP, 20 April 2020) (see Table 4).

**Table 4:** Italian opinion on psychological assistance during the pandemic.

**Question 1. In your opinion, how important is it, in this emergency, that psychological assistance is guaranteed by the public system?**			
Very Important (37%)	Quite Important (42%)	Total 79%	
Very + Quite important	Men 75%	Women 83%	
**Question 2. In which health care facilities should psychological assistance be used?**			
Healthcare facilities	Total	Men	Women
Hospital	90%	87%	93%
Nursing home for the elderly	87%	85%	89%
Social services	84%	83%	86%
To help family doctors	79%	75%	83%

*The survey was carried out by the Piepoli Institute for the National Council of the Order of Psychologists (April 20, 2020) on a total sample of 1,004 subjects representative of the Italian population aged 18 and over, segmented by gender*.

## The Need for More Mental Health to Achieve More Global Health

About 14% of the global burden of disease has been attributed to neuropsychiatric disorders, mainly represented by severe depression, substance abuse disorders, and psychoses (69). However, mental disorders increase the risk of communicable and non-communicable diseases just as many physical problems increase the risk of mental disturbances. Physical consequences of mental illness and vice versa are often neglected by health care system policies worldwide, affecting the quality of mental health services with direct consequences on the grade of disability and prognosis of people with mental illness. The pandemic worsened health care accessibility and safety, particularly for people suffering from mental diseases who were prone to higher health risks and mortality (17, 35, 70) and who experienced more frequent psychiatric symptoms during the lockdown periods than the general population (71). Specific efforts and intense preventive interventions, thus, might be urgently dedicated to this category of people.

### The Bidirectional Associations Between COVID-19 and Psychiatric Disorder

Wang et al. (72) analyzes a nationwide database of electronic health records of 61 million adult patients from 360 hospitals and 317,000 providers across 50 states in the United States, up through July 29, 2020, and compares COVID-19 infection rates among people with and without a recent psychiatric diagnosis. The analysis shows a higher risk of COVID-19 infection among people with recent psychiatric illness with a more than 7-fold increase in risk for people with depression and schizophrenia diagnoses. Increased risk was observed also for diagnoses of bipolar disorder and attention-deficit/hyperactivity disorder. There is also evidence of greater hospitalization and mortality rates among COVID-19 cases with a previous psychiatric history. Patients with both a recent diagnosis of a mental disorder and COVID-19 infection had a death rate of 8.5 vs. 4.7% among COVID-19 patients with no mental disorder and a hospitalization rate of 27.4% (vs. 18.6% among COVID-19 patients with no mental disorder, *p* < 0.001). Women were at higher risk than men.

The findings reported by Taquet et al. show a similar pattern. They compare 1,729,837 individuals with a psychiatric diagnosis in the previous year with a matched sample of the same size with no psychiatric history. There is a 65% increase in risk of COVID-19 for people with a recent psychiatric history. Older people (older than 75 years) were at higher risk with no differences observed between men and women.

Yang et al. (73) show that psychiatric hospitalization before the pandemic was associated with elevated odds of COVID-19 diagnosis (OR 1.44, 95% CI 1.28–1.62), COVID-19 inpatient hospitalization (1.55, 1.34–1.78), and COVID-19–related death (2.03, 1.59–2.59).

Hao et al. shows that levels of anxiety, depression, stress, and insomnia are higher in psychiatric patients during the pandemic and the lockdown, and psychiatric patients have more health concerns, impulsivity, and suicidal ideation with more than one third of them who fulfill the diagnostic criteria for PTSD. Poor physical health is also associated with higher levels of anxiety, depression, and stress-related symptoms.

Chevance et al. (74) identify four types of major vulnerabilities in patients suffering from mental disorders and who have to face the pandemic: (1) medical comorbidities that are more frequently found in psychiatric patients and that represent risk factors for severe COVID-19; (2) old age as an independent risk factor for both psychiatric diseases and COVID-19; (3) cognitive and behavioral troubles that can hamper compliance with confinement and hygiene measures; (4) psychosocial vulnerability due to stigmatization and/or socioeconomic difficulties.

As suggested by Steptoe (75), many COVID-19 symptoms are non-specific, and they might not be quickly recognized as originating from a SARS-CoV-2 infection, particularly by people with mental health problems who daily experience many physical symptoms. This could lead to a delay in recognition of the infection and in prompt therapeutic intervention, possibly resulting in rapid worsening of the disease. On the other hand, according to Steptoe's hypothesis, it is nevertheless possible to speculate that people with psychiatric problems are more likely to attend health care facilities where they could come into close contact with potentially infectious patients or with super-spreader hospital staff.

In the cited retrospective analysis by Taquet et al. (70), COVID-19 infection increases risk of psychiatric sequelae in the 3 months following diagnosis. COVID-19 patients have a greater likelihood of being diagnosed for the first time with a psychiatric disease compared with other subsequent health problems, such as influenza, upper respiratory tract infections, skin infections, bone fractures, and lithiasis diseases, with HR ranging from 1.3 to 2.5. The greatest risks were found for anxiety disorder, insomnia, and dementia.

### The Alliance Between Psychiatrists and Psychologists

In this time of pandemic emergency, mental health workers appear more vulnerable to work-related stress than other colleagues employed in different health care categories. A survey on mental health workers in Lombardy, the epicenter of the Italian COVID-19 epidemic, shows ~31% of the participants obtained a severe score in at least one of the burnout dimensions, 11.6% had moderate or severe levels of anxiety, and 6.6% had moderate or severe level of depression (76). Mental health workers' efforts to maintain the continuity of psychiatric care during the pandemic are challenging, especially in Italy where the number of psychiatrists, psychologists, and other figures (nurses, social workers, occupational therapists, rehabilitation counselors, and auxiliary staff) is chronically under-dimensioned. According to the 2018 report of the Italian Ministry of Health, the total number of psychiatrists and psychologists employed in Italian public and accredited mental health departments is 3.870 and 2.384, respectively (77). This dramatically small number of mental care specialists should be rapidly increased through specific health care policies in view of the predictable rise of the number of new diagnoses of post-COVID mental illness in the next months and years.

## Conclusions

The COVID-19 pandemic, which is at an exceptional level due to its interconnected implications on people's lives and well-being, on the functioning of health services, and on the economy of all nations, imposes the need to adopt a non-reductionist vision that does not examine and address the problems in a separate and parceled manner but that is capable of reading the complexity of the phenomenon. From this point of view, having a clear link between biomedical, psychological, and social aspects—which have already been highlighted by extensive evidence (32, 78, 79)—is essential if we want to overcome the challenge and allow for there to be a new start that can only take place on a new scientific and governance basis on a supranational scale.

To this end, we believe it is useful to participate in and support research programs that propose a multidisciplinary approach to the study of the pandemic and its multiple and interconnected global effects (80).

The studies on the Italian pandemic, the results of which we have analyzed, show a widespread state of psychological distress which, according to decades of scientific and clinical evidence on the relationship between mental states and immune system efficiency that we review in this work, plausibly weakens the resistance of individuals and the population to SARS-CoV-2 infection. Italy can deploy a great force, represented by tens of thousands of psychologists, psychotherapists, and psychiatrists, who could be employed, alongside local and hospital medicine, in primary care and in promoting the resilience of citizens and health workers themselves, who are subject to work stress that also includes a threat to their lives.

It is urgent to cement the alliance between medicine and psychology and between psychologists and psychiatrists with other mental health professionals in a framework of integration of mental health care to make real the unifying concept “no health without mental health” (69, 81) even during this threatening time.

## Data Availability Statement

The original contributions presented in the study are included in the article/supplementary material, further inquiries can be directed to the corresponding author/s.

## Author Contributions

All authors have contributed equally to the design, writing, and revision of the text.

## Conflict of Interest

The authors declare that the research was conducted in the absence of any commercial or financial relationships that could be construed as a potential conflict of interest.
